# Concomitant renal insufficiency and diabetes mellitus as prognostic factors for acute myocardial infarction

**DOI:** 10.1186/1475-2840-10-95

**Published:** 2011-10-31

**Authors:** Chang Seong Kim, Joon Seok Choi, Jeong Woo Park, Eun Hui Bae, Seong Kwon Ma, Myung Ho Jeong, Young Jo Kim, Myeong Chan Cho, Chong Jin Kim, Soo Wan Kim

**Affiliations:** 1 Department of Internal Medicine, Chonnam National University Medical School, Gwangju, Korea; 2 Cardiovascular Research Institute of Chonnam National University, Gwangju, Korea; 3 Department of Internal Medicine, Yeungnam University, Daegu, Korea; 4 Department of Internal Medicine, Chungbuk National University, Cheongju, Korea; 5 Department of Internal Medicine, Kyunghee University, Seoul, Korea

**Keywords:** acute myocardial infarction, diabetes mellitus, major adverse cardiac events, renal insufficiency

## Abstract

**Background:**

Diabetes mellitus and renal dysfunction are prognostic factors after acute myocardial infarction (AMI). However, few studies have assessed the effects of renal insufficiency in association with diabetes in the context of AMI. Here, we investigated the clinical outcomes according to the concomitance of renal dysfunction and diabetes mellitus in patients with AMI.

**Methods:**

From November 2005 to August 2008, 9905 patients (63 ± 13 years; 70% men) with AMI were enrolled in a nationwide prospective Korea Acute Myocardial Infarction Registry (KAMIR) and were categorized into 4 groups: Group I (n = 5700) had neither diabetes nor renal insufficiency (glomerular filtration rate [GFR] ≥ 60 ml/min/1.73 m^2^), Group II (n = 1730) had diabetes but no renal insufficiency, Group III (n = 1431) had no diabetes but renal insufficiency, and Group IV (n = 1044) had both diabetes and renal insufficiency. The primary endpoints were major adverse cardiac events (MACE), including a composite of all cause-of-death, myocardial infarction, target lesion revascularization, and coronary artery bypass graft after 1-year clinical follow-up.

**Results:**

Primary endpoints occurred in 1804 (18.2%) patients. There were significant differences in composite MACE among the 4 groups (Group I, 12.5%; Group II, 15.7%; Group III, 30.5%; Group IV, 36.5%; *p *< 0.001). In a Cox proportional hazards model, after adjusting for multiple covariates, the 1-year mortality increased stepwise from Group III to IV as compared with Group I (hazard ratio [HR], 1.96; 95% confidence interval [CI], 1.34-2.86; *p *= 0.001; and HR, 2.42; 95% CI, 1.62-3.62; *p *< 0.001, respectively). However, Kaplan-Meier analysis showed no significant difference in probability of death at 1 year between Group III and IV (p = 0.288).

**Conclusions:**

Renal insufficiency, especially in association with diabetes, is associated with the occurrence of composite MACE and indicates poor prognosis in patients with AMI. Categorization of patients with diabetes and/or renal insufficiency provides valuable information for early-risk stratification of AMI patients.

## Background

Renal dysfunction and diabetes mellitus are established risk factors for long-term adverse prognosis in patients with cardiovascular disease. Any degree of preexisting renal dysfunction should be considered a potent, independent risk factor for cardiovascular complication after acute myocardial infarction (AMI) [1,2]. The 1-year mortality after AMI is approximately 60% in end-stage renal disease (ESRD) [3], and renal dysfunction has been reported to be independently predictive of death after admission for acute coronary syndrome [4,5], clearly indicating that the risk of subsequent cardiovascular events in patients with renal dysfunction is higher than in subjects with normal renal function [6,7]. Notwithstanding the imminent risks, the mechanisms by which renal dysfunction causes cardiovascular disease remain unclear.

The prevalence of diabetes mellitus has rapidly increased worldwide. Strikingly, diabetes mellitus is (similar to coronary artery disease) a known risk factor for cardiovascular events such as myocardial infarction and cardiovascular death [8,9]. A large, prospective multinational registry, the Global Registry of Acute Coronary Events (GRACE) [10], revealed that in-hospital mortality of patients with diabetes concomitant with acute coronary syndrome is almost twice as high as that of patients without diabetes. Moreover, in a recent study [11], diabetes has been suggested as a significant independent risk factor for acute coronary syndrome.

Therefore, the presence of diabetes mellitus and renal dysfunction may individually or simultaneously have a negative prognostic effect on patients with AMI. Yet several studies have evaluated mortality in the presence or absence of diabetes and renal insufficiency after AMI [12,13], limited information exists on the role of renal insufficiency and its association with diabetes mellitus in the context of AMI. The aim of the present study was to investigate the clinical outcomes according to the concomitance of renal dysfunction and diabetes mellitus in patients with AMI.

## Methods

### Study design and patient population

The study population was enrolled in a nationwide prospective Korea Acute Myocardial Infarction Registry (KAMIR) from November 2005 to August 2008. This study was a retrospective cohort of 9905 consecutive patients (mean age ± standard deviation [SD], 63 ± 13 years; 70% men) whose discharge diagnosis was AMI based on clinical symptoms, cardiac enzyme levels, and 12-lead electrocardiogram [14]. This study included patients who were available to calculated estimated GFR. The patients with underlying malignancy were excluded. All of the patients completed at least 1 year of follow-up.

The KAMIR, launched in November 2005, is a Korean prospective multicenter data collection registry reflecting real-world treatment practices and outcomes in Asian patients diagnosed with AMI. The registry consists of 52 community and university hospitals with facilities for primary percutaneous coronary intervention (PCI), thrombolytic therapy and on-site cardiac surgery. Data were collected by a well-trained study coordinator based on a standardized case report form and protocol. The study protocol was approved by the ethics committee at each participating institution and all patients were informed about their participation in this registry.

For simplicity of analysis and presentation, all patients were categorized into 4 groups according to the presence of diabetes mellitus and renal insufficiency (glomerular filtration rate [GFR] < 60 ml/min/1.73 m^2^). Group I (n = 5700) had neither diabetes mellitus nor renal insufficiency (GFR ≥ 60 ml/min/1.73 m^2^); Group II (n = 1730) had diabetes mellitus but no renal insufficiency; Group III (n = 1431) had no diabetes mellitus but renal insufficiency; Group IV (n = 1044) had both diabetes mellitus and renal insufficiency.

### Definition

Diabetes mellitus was defined as fasting plasma glucose level of 126 mg/dl or greater on at least 2 occasions, plasma glucose of 200 mg/dl or greater at 2 h after a 75-g oral glucose tolerance test, the need for insulin or glucose-lowering medication to control glucose levels on admission, or medical history of diet-controlled diabetes. Among AMI patients, ST-segment elevation acute myocardial infarction (STEMI) was defined as new ST-segment elevation of > 2 mm in ≥ 2 pre-cordial leads or > 1 mm in ≥ 2 limb leads, or a new onset of left bundle branch block on the 12-lead electrocardiogram with a concomitant increase of at least one cardiac biomarker of necrosis (e.g., creatine kinase-MB, troponin I, or troponin T). Non-ST-segment elevation acute myocardial infarction (NSTEMI) was defined as the exception of STEMI. Left ventricular ejection fraction (LVEF) was checked by two-dimensional echocardiography.

### Assessment of renal function

Renal insufficiency was defined as an estimated GFR of < 60 ml/min/1.73 m^2 ^calculated using the Modification of Diet in Renal Disease (MDRD) [15] formula, including age, ethnicity, sex, and serum creatinine: GFR, in ml/min per 1.72 m^2 ^= 1.86 × (serum creatinine [ml/min])^-1.154 ^× (age)^-0.203 ^× (0.742 [for women]). Serum creatinine was analyzed by the alkaline picrate method performed using an Olympus 5431^® ^device (Olympus Optical Co. Ltd., Tokyo, Japan). The level of creatinine was measured prior to angiography, and renal function was assessed based on the estimation of GFR.

### Study endpoint

The primary endpoints were major adverse cardiac events (MACE), including a composite of all cause-of-death, myocardial infarction, target lesion revascularization, and coronary artery bypass graft during the 12-month clinical follow-up. Target lesion revascularization was defined as any revascularization of the target lesion because of restenosis or reocclusion within the stent or adjacent 5-mm border.

### Statistical analysis

Continuous variables are presented as means ± SD, and categorical variables as number of cases and percentages. Comparative analysis was performed within the groups or as a whole using either analysis of variance (ANOVA) or Student's *t*-test for continuous variables, and Pearson chi-square test or Fisher's exact test for categorical variables. Continuous variables with skewed distribution were presented as median (with 25th and 75th percentiles) and compared by using the Kruskal-Wallis test. Logistic regression was performed to identify the independent predictors of MACE at 1-year clinical follow-up. Multivariate Cox regression analysis was adjusted by previous medical knowledge and independently of *p *value: age, sex, body mass index, systolic blood pressure on admission, heart rate, Killip class > I, history of hypertension, dyslipidemia, coronary artery disease, smoking, multivessel disease, LVEF < 55%, medication of statin, low-density lipoprotein cholesterol, and N-terminal pro-brain natriuretic peptide (NT-pro BNP) of > 3000 pg/ml. The probability of death was estimated by the Kaplan-Meier method, and curves were compared with the log-rank test. All statistical tests were 2-tailed, and *p *< 0.05 was considered significant. Analyses were performed using the Statistical Package for Social Sciences software, version 17.0 (SPSS, Chicago, Illinois).

## Results

### Baseline characteristics of the study patients

A total of 9905 patients with AMI were included in the present study. The baseline characteristics of the patients are listed in Table 1. Patients from Group I toward Group IV were increasingly older, more frequently women, had higher rates of previous hypertension episodes and coronary artery disease, were above Killip class I, and exhibited increasing NT-pro BNP and high-sensitivity C-reactive protein (hs-CRP). On the contrary, the rate of smoking history, family history of coronary artery disease, low-density lipoprotein cholesterol levels, and LVEF decreased from Group I toward Group IV. There were significant differences in heart rate, systolic and diastolic blood pressure, previous dyslipidemia, and presence of STEMI or NSTEMI among the groups. However, there were no differences in body weight or body mass index. Group IV had significantly higher rate of insulin treatment compared to Group II, but there were no differences in oral hypoglycemic agents between the 2 groups. In-hospital medications are also listed in Table 1. It is noteworthy that Group IV had higher levels of creatinine and lower estimated GFR.

**Table 1:** Baseline clinical characteristics

	Group I (n = 5700)	Group II (n = 1730)	Group III (n = 1431)	Group IV (n = 1044)	*p *Value	
					
					Overall	Linear†
Age (years)	61 ± 13	62 ± 11^¶^	71 ± 11	70 ± 9^‡§^	< 0.001	
Age groups (years)					< 0.001	< 0.001
< 65	2671(47%)	706(41%)	231(16%)	136(13%)		
65-74	2165(38%)	781(45%)	598(42%)	586(56%)		
≥ 75	864(15%)	243(14%)	602(42%)	322(31%)		
Male (%)	4411(77)	1222(71)^¶^	812(57)	534 (51)^‡§^	< 0.001	< 0.001
Body weight (kg)	65 ± 12	66 ± 12^¶^	61 ± 12	62 ± 11	0.095	
Body mass index (kg/m^2^)	24 ± 3	25 ± 4^¶^	23 ± 3	24 ± 3^§^	0.483	
HR(beats/minute)	76 ± 20	80 ± 20^¶^	79 ± 31	83 ± 26^‡§^	< 0.001	
Systolic blood pressure(mm Hg)	130 ± 27	130 ± 28	121 ± 35	127 ± 34^‡^	< 0.001	
Diastolic blood pressure(mm Hg)	80 ± 19	79 ± 16	74 ± 21	76 ± 35^‡^	< 0.001	
Current smoker (%)	3690(65)	990(58)^¶^	660(46)	380(37)^‡§^	< 0.001	< 0.001
Treatment of Diabetes mellitus						
No treatment (%)	5700(100)	260(15.2)	1431(100)	65(7)^‡^		
Insulin treatment (%)		101(5.8)		160(15)^‡^		
Oral hypoglycemic agents (%)		1369(79)		819(78)		
Previous hypertension (%)	2168(38)	987(57)^¶^	863(60)	772(74)^‡§^	< 0.001	< 0.001
Previous dyslipidemia (%)	497(9)	212(12)^¶^	118(8)	133(13)^§^	< 0.001	0.001
Family history of CAD (%)	446(8)	118(7)	63(4)	43(4)^‡^	< 0.001	< 0.001
Previous CAD (%)	684(12)	311(18)^¶^	293(21)	273(26)^‡§^	< 0.001	< 0.001
Killip class > I (%)	1099(20)	423 (25)^¶^	603 (43)	508 (50)^‡§^	< 0.001	< 0.001
LDL-C (mg/dl)	120 ± 40	114 ± 38^¶^	112 ± 43	110 ± 52^‡^	< 0.001	
NT-proBNP (pg/ml)	323 (81, 1151)	468 (123, 1577)	1750 (338, 6804)	3522 (877, 10894)	< 0.001	
hs-CRP	0.64 (0.17, 3.53)	0.92 (0.23, 4.15)	1.46 (0.30, 6.59)	2.19 (0.36, 9.70)	< 0.001	
LVEF (%)	53 ± 12	51 ± 12^¶^	49 ± 14	46 ± 14^‡§^	< 0.001	
STEMI (%)	3619(64)	1009(58)^¶^	908(63)	518(50)^‡§^	< 0.001	
Non-STEMI (%)	2083(37)	722(42)^¶^	524(37)	526(50)^‡§^	< 0.001	
Kidney function						
Serum Creatinine (mg/dL)	0.92 ± 0.19	0.91 ± 0.19^¶^	2.08 ± 3.58	2.38 ± 3.44^‡§^	< 0.001	
Estimated GFR* (ml/min/1.73 m^2^)	88.9 ± 39.2	87.5 ± 32.9	44.7 ± 14.2	39.0 ± 15.9^‡§^	< 0.001	
Medications (%)						
Aspirin	5616 (99)	1706 (99)	1381 (97)	1009(97)^‡^	< 0.001	< 0.001
Clopidogrel	5523(97)	1675(97)	1340(94)	968(93)^‡^	< 0.001	< 0.001
Beta blocker	4295 (76)	1337 (78)	908 (64)	694 (67)^‡^	< 0.001	< 0.001
CCB	707(12)	215(13)	205(14)	208(20)^‡§^	< 0.001	< 0.001
Statin	4274 (75)	1295 (75)	906 (64)	667 (64)^‡^	< 0.001	< 0.001
ACE inhibitor	4191 (74)	1294 (75)	891 (63)	629 (60)^‡^	< 0.001	< 0.001
ARB	656 (12)	249 (14)^¶^	235 (17)	213 (21)^‡§^	< 0.001	< 0.001

Data are presented as mean ± SD or number of patients (percentage). Not normally distributed variables were described as median (25^th^, 75^th ^percentile). Group I, estimated GFR ≥ 60(ml/min/1.73 m^2^) without DM; Group II, estimated GFR ≥ 60 with DM; Group III, estimated GFR < 60 without DM; Group IV, estimated GFR < 60 with DM.

Abbreviations: HR, heart rate; CAD, coronary arterial disease; LDL-C, low-density lipoprotein cholesterol; NT-proBNP, N-terminal pro-brain natriuretic peptide; hs-CRP, high-sensitivity C-Reactive Protein; LVEF, left ventricular ejection fraction; STEMI, ST-segment elevation acute myocardial infarction; GFR, Glomerular filtration rate; CCB, calcium channel blocker; ACE, angiotensin converting enzyme; ARB, angiotensin II receptor blockers.

*On the basis of abbreviated MDRD (Modification of Diet in Renal Disease) study equation.

†Statistical significance for linear-by-linear association between categorical variables calculated using chi-square-test for trend.

¶ *p *< 0.05 Compared with Group I.

‡*p *< 0.05 Compared with Group II.

§ *p *< 0.05 Compared with Group III.

### Angiographic and procedural characteristics

The coronary angiographic and procedural characteristics are listed in Table 2. The number of involved coronary arteries was increasingly higher in patients of Group I through IV. The number of complex lesions (B2 and C) as defined by the American College of Cardiology/American Heart Association (ACC/AHA) was also increasingly higher. Moreover, post-procedure thrombolysis in myocardial infarction (TIMI) flow was lower in patients of Group IV as compared with Group I.

**Table 2:** Angiographic and procedural characteristics

	Group I (n = 5700)	Group II (n = 1730)	Group III (n = 1431)	Group IV (n = 1044)	*p *Value	
					
					Overall	Linear†
Location of culprit coronary lesion (%)						
Left anterior descending	2599(50)	759(47)^¶^	500(42)	365(42)^‡^	< 0.001	< 0.001
Left circumflex	888(17)	271(17)	179(15)	143(17)	0.354	0.208
Right	1639(32)	544(34)	480(40)	326(38)^‡^	< 0.001	< 0.001
Left main	77(2)	40(3)^¶^	40(3)	35(4)^‡^	< 0.001	< 0.001
No. of coronary arteries narrowed (%)						
One vessel	2552(49)	580(36)^¶^	407(34)	203(23)^‡§^	< 0.001	< 0.001
Two vessel	1524(29)	512(32)	388(32)	244(28)^‡§^	0.039	0.543
Three vessel	998(19)	455(28)^¶^	354(30)	372(43)^‡§^	< 0.001	< 0.001
Multi-vessel	2645(51)	1041(64)^¶^	789(66)	673(77)^‡§^	< 0.001	< 0.001
ACC/AHA lesion score (%)						
A	235 (5)	63(4)	49(4)	35(4)	0.679	0.342
B1	855(18)	283(19)	174(16)	126(15)^‡^	0.087	0.078
B2	1384(28)	449(30)	262(23)	204(25)^‡^	0.001	0.002
C	2415(49)	724(48)	635(57)	455(56)^‡^	< 0.001	< 0.001
Post-procedure TIMI flow ≥ III (%)	4541(93)	1394(93)	970(88)	689(88)^‡^	< 0.001	< 0.001

Groups are as in Table 1.

Abbreviations: ACC/AHA, American College of Cardiology/American Heart Association; TIMI, thrombolysis in myocardial infarction.

†Statistical significance for linear-by-linear association between categorical variables calculated using chi-square-test for trend.

¶ *p *< 0.05 Compared with Group I.

‡*p *< 0.05 Compared with Group II.

§ *p *< 0.05 Compared with Group III.

### In-hospital mortality and clinical outcomes during follow-up

**Figure 1:**
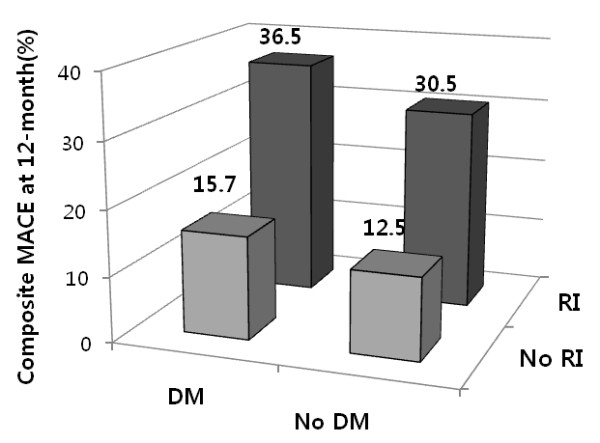
**Composite MACE at 12-month stratified by the presence diabetes mellitus (DM) and renal insufficiency (RI)**.

**Figure 2:**
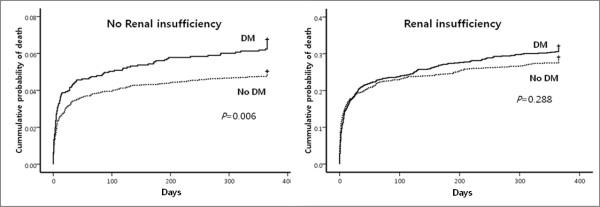
**Kaplan-Meier curves according to renal insufficiency in patients with or without diabetes mellitus (DM)**.

Clinical outcomes in hospital and at 1 month and 12 months after discharge are listed in Table 3. Group IV had a significantly higher incidence of composite MACE, myocardial infarction, and death after 1-month and 12-month clinical follow-up. There was a stepwise increase in 12-month composite MACE in patients of Group I through IV (Figure 1). However, target lesion revascularization at 1 month and coronary artery bypass graft at 12 months were not significantly different among the groups. In patients with no renal insufficiency (Groups I and II), those with diabetes had significantly more 12-month composite MACE than those without diabetes (12.5% versus 15.7%, respectively; *p *= 0.001). In patients with renal insufficiency (Groups III and IV), the same was observed (30.5% versus 36.5%, respectively; *p *= 0.002). However, there were no significant differences between diabetic and non-diabetic patients with regard to 12-month mortality in the renal insufficiency groups (25.3% in Group III versus 27.5% in Group IV; *p *= 0.212). Thus, both myocardial infarction and target lesion revascularization might be affecting the increase of 12-month composite MACE in diabetic patients with renal insufficiency. Kaplan-Meier curve analysis (Figure 2) revealed that patients with diabetes had significantly higher death rates than those without diabetes under no renal insufficiency conditions (*p *= 0.006). The same was not observed in diabetic and non-diabetic patients with renal insufficiency (*p *= 0.288).

**Table 3:** Clinical outcomes in-hospital period and follow up.

	Group I (n = 5702)	Group II (n = 1731)	Group III (n = 1432)	Group IV (n = 1044)	*p *Value	
					
					Overall	Linear†
In-hospital outcomes (n = 9905)						
In-hospital death (%)	168(3)	66(4)	249(18)	185(18)^‡^	< 0.001	< 0.001
1-month outcomes						
Composite MACE (%)	327 (5.7)	128(7.4)^¶^	317(22.2)	257(24.6)^‡^	< 0.001	< 0.001
Death (%)	225(3.9)	84(4.9)	296(20.7)	226(21.6)^‡^	< 0.001	< 0.001
MI (%)	28(0.5)	12(0.7)	4(0.3)	19(1.8)^‡^	< 0.001	0.001
Re-PCI (%)	57(1.0)	21(1.2)	16(1.1)	7(0.7)	0.559	0.649
CABG (%)	17(0.3)	11(0.6)	1(0.1)	5(0.5)	0.039	0.807
12-month outcomes						
Composite MACE (%)	715 (12.5)	271 (15.7)^¶^	436 (30.5)	381 (36.5)^‡§^	< 0.001	< 0.001
Death (%)	287(5.0)	117 (6.8)^¶^	361 (25.3)	287 (27.5)^‡^	< 0.001	< 0.001
MI (%)	44(0.8)	20 (1.2)	10 (0.7)	25 (2.4)^‡§^	< 0.001	< 0.001
Re-PCI (%)	385(6.3)	120 (6.9)	59 (4.1)	62 (5.9)^§^	0.006	0.082
CABG (%)	26 (0.5)	14 (0.8)	6 (0.4)	7 (0.7)	0.280	0.455

Groups are as in Table 1.

Abbreviations: MACE, major adverse cardiac event; MI, myocardial infarction; Re-PCI, target lesion revascularization; CABG, coronary artery bypass graft.

†Statistical significance for linear-by-linear association between categorical variables calculated using chi-square-test for trend.

¶ *p *< 0.05 Compared with Group I.

‡*p *< 0.05 Compared with Group II.

§ *p *< 0.05 Compared with Group III.

### Cox regression analysis for mortality during follow-up

Multivariable Cox regression analysis was performed to identify the risk factor(s) accounting for the correlation effect of renal insufficiency and diabetes in 12-month mortality. The results of a multivariable Cox proportional hazards model adjusting for other potential clinical predictors of mortality are shown in Table 4. After adjusting for multiple covariates, Group II (diabetes and no renal insufficiency) showed no significant differences in 12-month mortality as compared with Group I (no diabetes and no renal insufficiency) (hazard ratio [HR], 1.20; 95% confidence interval [CI], 0.86-2.02; *p *= 0.209). However, the 12-month mortality increased stepwise from Group III to Group IV as compared with Group I (HR, 1.96; 95% CI, 1.34-2.86; *p *= 0.001; and HR, 2.42; 95% CI, 1.62-3.62; *p *< 0.001, respectively).

**Table 4:** Prognostic values of combined use of renal insufficiency and diabetes mellitus for 12 months mortality (Cox proportional hazards model)

	Hazard Ratio (95% confidence interval)			
	
	No adjustment	*P *value	Adjustment	*P *value
Group I**				
Group II	1.35 (1.09-1.68)	0.006	1.32 (0.86-2.02)	0.209
Group III	5.65 (4.84-6.60)	< 0.001	1.96 (1.34-2.86)	0.001
Group IV	6.14 (5.21-7.23)	< 0.001	2.42 (1.62-3.62)	< 0.001

**Reference group

Group I, estimated GFR ≥ 60(ml/min/1.73 m^2^) without DM; Group II, estimated GFR ≥ 60 with DM; Group III, estimated GFR < 60 without DM; Group IV, estimated GFR < 60 with DM. Adjusted for factors included in age, sex, body mass index, systolic blood pressure on admission, heart rate, Killip class > I, history of hypertension, dyslipidemia, coronary artery disease, smoking, multi vessel disease, LVEF < 55%, medication of statins, low-density lipoprotein cholesterol, NT-pro BNP of > 3000 (pg/ml).

### Independent predictors of major adverse cardiac events

We performed multivariate logistic regression analysis to identify the independent predictors of MACE. Predictors of 12-month MACE were Killip class > I, diabetes mellitus, previous coronary artery disease, decrease in LVEF by < 55%, estimated GFR of < 60 ml/min/1.73 m^2^, and increase in levels of NT-pro BNP by > 3000 pg/ml. On the other hand, in-hospital prescription of beta-blockers and statins were negative predictors of 12-month MACE (Table 5).

**Table 5:** Independent predictors of One-year Major Adverse Cardiac Events.

	Odd ratio (95% confidence interval)	*P *value
Killip > I	1.48(1.24-1.76)	< 0.001
Hypertension	1.04(0.89-1.23)	0.616
Diabetes mellitus	1.26(1.03-1.45)	0.019
Dyslipidemia	0.84(0.64-1.10)	0.197
Previous CAD	1.49(1.22-1.82)	< 0.001
History of smoking	1.01(0.86-1.18)	0.931
Familial history of CAD	1.01(0.74-1.38)	0.949
LVEF < 55%	1.23(1.04-1.46)	0.017
Estimated GFR* < 60 (ml/min/1.73 m^2^)	1.45(1.21-1.75)	< 0.001
NT-proBNP > 3000 (pg/ml)	2.17(1.79-2.64)	< 0.001
Aspirin	0.63(0.28-1.45)	0.281
Beta blocker	0.72(0.59-0.88)	0.001
ACE inhibitor	0.92(0.75-1.12)	0.393
ARB	0.85(0.67-1.08)	0.184
Statin	0.80(0.67-0.95)	0.012

*On the basis of abbreviated MDRD (Modification of Diet in Renal Disease) study equation.

Abbreviations: CAD, coronary arterial disease; LVEF, left ventricular ejection fraction; GFR, Glomerular filtration rate; NT-proBNP, N-terminal pro brain natriuretic peptide; ACE, angiotensin converting enzyme; ARB, angiotensin II receptor blockers.

## Discussion

This study was designed to investigate the clinical outcomes of patients with AMI as function of the presence or absence of renal dysfunction and diabetes. Many clinical studies have been conducted on the association between renal dysfunction and mortality among patients with AMI [1,2,6,16] or have evaluated the influence of diabetes on mortality following acute coronary syndrome [11,17-20]. In fact, renal dysfunction and diabetes are associated with adverse clinical outcomes after AMI. However, limited information exists with regard to cardiovascular disease risk in patients with diabetes and renal dysfunction, particularly after AMI. It is also unknown whether the relation between diabetes and cardiovascular outcomes differs between patients with or without renal dysfunction.

### Correlation between clinical outcomes and presence of renal insufficiency and diabetes in patients with AMI

Our findings confirm those of previous studies [1,2,6,16] that deteriorating renal function predicts mortality after AMI. In our study, renal insufficiency groups (Group III and IV) had higher composite MACE occurrence and mortality after a 12-month follow-up as compared with groups with no renal insufficiency. There are several potential links between renal insufficiency and increased number of cardiovascular events. First of all, renal insufficiency is accompanied by anemia, high level of homocysteine, increased oxidation of low-density lipoproteins, and diminished nitric oxide production, which may result in accelerated atherosclerosis, endothelial dysfunction, and poor outcome after myocardial infarction [21-23]. In addition, left ventricular hypertrophy accompanying advanced renal failure is an important risk factor for mortality resulting from cardiovascular disease. In the clinical setting, renal insufficiency might be another useful surrogate marker for cardiovascular disease in the patients with diabetes, because the estimated GFR was associated with both the intima-media thickness and the brachial-ankle pulse wave velocities in previous study [24]. Moreover, diabetic patients with non-albuminuric renal impairment had higher prevalence of cardiovascular disease than those with albuminuria and non-reduced estimated GFR [25-27]. Therefore, renal impairment is a powerful predictor of cardiovascular disease morbidity and mortality in diabetic patients. Similarly, in our study, angiographic findings such as left main coronary artery disease and ACC/AHA lesion scores were more severe in renal insufficiency than in non-renal insufficiency groups.

Diabetic patients with AMI hold poorer clinical outcomes than non-diabetic patients [19]. Our findings supported the observation that patients with diabetes have more composite MACE at 1 year of follow-up than those without diabetes, regardless of renal insufficiency. Multiple mechanisms have been implicated in the increased number of adverse outcomes in diabetes patients. These mechanisms include an abnormal metabolic response to ischemia with inefficient energy use and accumulation of deleterious oxygen-free radicals [28], greater endothelial dysfunction [29], and abnormalities of thrombosis and fibrinolysis [30]. Recently, Yan *et al*. [31] demonstrated a significant association between plasma osteopontin levels and the presence and severity of coronary artery disease in diabetic patients, indicating that osteopontin may be critically involved in the inflammatory processes resulting in accelerated atherosclerosis. Patients with diabetes are known to have a greater atherosclerosis burden, with more diffuse and more multivessel coronary artery disease, as seen in our study.

Taking all our findings together, we suggest that concomitant occurrence of renal insufficiency and diabetes in patients with AMI represent not only severity of disease or clinical outcome, but also serve as prognostic predictor of cardiovascular risk, including composite MACE and all-cause mortality.

### Clinical relevance of the classification according to renal insufficiency and diabetes in patients with AMI

The present study demonstrated that simply categorizing patients into 4 groups according to the presence and absence of diabetes and renal insufficiency was efficient in differentially predicting 1-year clinical outcome after AMI. Despite no significant differences between Group I and Group II in multivariate Cox regression analysis, we found a stepwise increase in the HR for 12-month mortality from Group I through Group IV. These observations were further supported by the fact that patients in higher groups were older, had a higher prevalence of hypertension and previous coronary artery disease, and lower LVEF compared with Group I, all of which may induce atherosclerosis and contribute to increased cardiovascular mortality. In this respect, we also found that several molecular markers, including NT-pro BNP and hs-CRP were increased from Group I through IV. Such markers are affected not only by the degree of heart failure, but also by inflammation [32,33], which might have increased the risk of cardiovascular mortality in our patients with AMI. Therefore, this study suggests that categorization of patients according to the presence of diabetes and renal insufficiency provides valuable information for early-risk stratification of patients with AMI.

Recently, a single-center prospective study [13] encompassing 3334 AMI patients divided into those without diabetes, with diabetes and chronic kidney disease (CKD), and with diabetes and without CKD, suggested that mortality and MACE rates did not differ significantly between diabetes patients without CKD and patients without diabetes. Diabetes coexisting with CKD, however, was found to be one of the strongest independent risk factors for cardiovascular complications and total mortality, in agreement with our findings. On the other hand, some differences in the clinical outcomes were observed in our study. The non-diabetic patients were divided into 2 groups according to presence or absence of renal insufficiency, and non-diabetic patients with renal insufficiency (Group III) had higher mortality and 12-month composite MACE than diabetic patients without renal insufficiency (Group II). On the contrary, patients without diabetes and renal insufficiency (Group I) had less severe adverse clinical outcomes following AMI than diabetic patients without renal insufficiency (Group II).

Anavekar *et al*. [12] showed that patients with or without diabetes present a similar relationship between renal insufficiency and cardiovascular risk, including all-cause mortality, death, a composite of heart failure, recurrent AMI, resuscitated sudden cardiac death, and stroke, after high-risk AMI. In agreement with a previous study [12], our study demonstrated that the mortality at 12 months of follow-up was not different between diabetic and non-diabetic patients with renal insufficiency after AMI. This indicates that patients with renal dysfunction might be more susceptible to severe and diffuse coronary artery disease, regardless of diabetes. Indeed, such patients present a high prevalence of left ventricular hypertrophy and hypertension, both of which are markers of increased cardiovascular risk [34,35].

Although patients with and without diabetes experience increased cardiovascular event rates with worsening of renal function, patients with diabetes consistently show higher proportions of events than non-diabetic patients according to decreasing renal function [12]. Consequently, diabetes and renal insufficiency independently or synergistically are related with an increase in cardiovascular events after AMI. Therefore, we underline the importance of classifying patients into 4 large groups according to presence or absence of diabetes and renal insufficiency for predicting mortality and cardiovascular complications in patients following AMI.

### Study limitations

Nonetheless, the present study has several limitations. The best validated method to calculate estimated GFR is the MDRD formula. However, this formula has not been specifically validated in patients with diabetes [36]. With respect to increasing GFR in patients with early diabetes, the influence of GFR changes was not addressed. In addition, even healthy but more than 65 years old female subjects can reach a GFR of less than 60 ml/min/1.73 m^2^, indicating that the definition of renal insufficiency is weak. Secondly, since our study was retrospective and non-randomized, it may reflect selection bias. Third, although most confounders were included in the multivariate analysis, it is possible that some confounders were excluded. Finally, clinical data encompassed a 1-year period in our study. Thus, long-term clinical outcomes could not be fully evaluated. Large-scale long-term prospective randomized trials are needed in the future.

## Conclusions

Renal insufficiency, especially in association with diabetes, is associated with the occurrence of composite MACE and is a predictor of poor prognosis in patients with AMI. Categorization of patients according to presence or absence of diabetes and renal insufficiency provides valuable information for early-risk stratification of patients with AMI.

## Abbreviations

ACC/AHA: American College of Cardiology/American Heart Association; AMI: acute myocardial infarction; CI: confidence interval; CKD: chronic kidney disease; ESRD: end-stage renal disease; GFR: glomerular filtration rate; GRACE: Global Registry of Acute Coronary Events; HR: hazard ratio; hs-CRP: high-sensitivity C-reactive protein; KAMIR: Korea Acute Myocardial Infarction Registry; LVEF: left ventricular ejection fraction; MACE: major adverse cardiac events; MDRD: Modification of Diet in Renal Disease; NSTEMI: non-ST-segment elevation acute myocardial infarction; NT-pro BNP: N-terminal pro-brain natriuretic peptide; PCI: percutaneous coronary intervention; STEMI: ST-segment elevation acute myocardial infarction; TIMI: thrombolysis in myocardial infarction.

## Competing interests

The authors declare that they have no competing interests.

## Authors' contributions

CSK carried out the research design, performed the statistical analysis and final preparation of the manuscript. JSC contributed in its design, performance. JWP and EHB participated in the design of this study. SKM contributed in the research design and result interpretation. MHJ, YJK, MCC and CJK participated in Korea Acute Myocardial Infarction Registry (KAMIR). SWK contributed in the research design, results interpretation and final preparation of the manuscript. The KAMIR investigators furnished valuable data. All authors have read and approved the final manuscript.
